# Editorial: Exploring the Therapeutic Potential of Natural Products in Internal Diseases

**DOI:** 10.3390/ph18111667

**Published:** 2025-11-04

**Authors:** Dorin Dragos, Adelina Vlad

**Affiliations:** 1 Department of Medical Semiology, Faculty of Medicine, Carol Davila University of Medicine and Pharmacy, 020021 Bucharest, Romania; 2 Department of Functional Sciences I/Physiology and Neuroscience, Faculty of Medicine, Carol Davila University of Medicine and Pharmacy, 050474 Bucharest, Romania

In recent decades, significant progress in drug development has transformed the treatment of internal diseases, improving both outcomes and prognosis [1]. However, these advances frequently come with a cost: serious, sometimes even life-threatening side effects [2]. Many clinicians acknowledge the need for alternatives but often lack the knowledge or confidence to use them. Meanwhile, an increasing number of people are resorting to complementary or alternative approaches, particularly herbal medicines [3,4]; these individuals have begun adopting healthier lifestyle changes alongside these treatments [5]. Despite this trend, skepticism toward natural products remains strong in mainstream medicine, largely due to the limited amount of high-quality evidence [3,4].

The Special Issue *“Therapeutic Potential of Natural Products in Internal Diseases”* was established to address the above challenges. It presents a collection of original research and critical reviews exploring how natural products might contribute to treating these conditions. Together, the studies included identify both the opportunities and the obstacles in translating traditional knowledge and early findings into evidence-based therapies.

With regard to metabolic disorders, several studies have illustrated the complexity of natural-product interventions. Bekheit et al. demonstrated that ferulic acid attenuated isoproterenol-induced myocardial infarction in diabetic rats, normalizing ECG patterns, lowering cardiac enzymes, and reducing oxidative stress and inflammatory cytokines by suppressing the AGE–RAGE axis [6]. Complementing this mechanistic insight, after conducting a before-and-after trial, Mantadaki et al. reported that quercetin supplementation (500 mg/day) over two 12-week periods increased the whole-blood absolute telomere length in type 2 diabetes patients, indicating potential anti-aging and cardiometabolic benefits [7]. A randomized, double-blind, placebo-controlled trial by Hariharan et al. examined carnosine supplementation (2 g/day) over 14 weeks in adults with prediabetes or well-controlled type 2 diabetes [8]. Although the intervention was safe and well-tolerated, it did not improve cognitive outcomes such as executive function or memory. The findings emphasize the importance of larger, longer trials targeting higher-risk or cognitively impaired populations to determine whether carnosine may offer benefit in metabolic disease.

Non-alcoholic fatty liver disease (NAFLD), especially in lean individuals, is being increasingly recognized as a distinct clinical challenge. Tang et al. investigated Ganjianglingzhu decoction, a traditional herbal formula, in a murine model of lean NAFLD [9]. The intervention ameliorated hepatic steatosis, inflammation, fibrosis, and oxidative stress, with metabolomics pointing to glycerophospholipid metabolism as a key mechanism. Specific metabolites such as sn-3-O-(geranylgeranyl)glycerol 1-phosphate emerged as potential biomarkers of effect, emphasizing how modern metabolomics can unlock new pathways in traditional medicine research.

Inflammation is a common thread in many chronic diseases, and plant-derived compounds demonstrate considerable promise as modulators of inflammatory processes. Du et al. revealed that the traditional Chinese medicine formula Qilianxiaopi reduced inflammation in models of chronic atrophic gastritis by targeting ADAM17 and lowering TNF-α secretion, with narirutin identified as a key active component [10]. Karinchai et al. reported that extracts from *Ficus lindsayana* roots and latex suppressed inflammation and improved adipocyte function in vitro, with effects on glucose uptake, lipolysis, and inflammatory cytokine expression [11]. These findings highlight the potential of herbal medicines to influence the intersection of inflammation and metabolic regulation.

Natural products are also garnering attention for their anticancer potential. Aziz et al. characterized methanol extracts of *Ammi visnaga* roots and seeds, showing antioxidant, antimicrobial, and cytotoxic activity against breast cancer cell lines, accompanied by an upregulation of pro-apoptotic genes and a downregulation of anti-apoptotic genes [12]. Pristimerin, a triterpenoid found in Celastraceae and Hippocrateaceae plants, has been highlighted by Prabhu et al. for its ability to inhibit cancer cell proliferation, induce apoptosis, and suppress metastasis through multiple signaling pathways [13]. Despite promising preclinical evidence, the authors recommend further clinical trials to confirm the therapeutic value of pristimerin.

Taken together, these contributions illustrate the therapeutic potential of natural products in addressing a wide spectrum of internal diseases, from metabolic and inflammatory disorders to liver disease and cancer. They underline the need for rigorous, mechanistic, and clinical investigations to transform traditional and preclinical insights into safe, effective, and evidence-based therapies. This Special Issue stands as a call to action for researchers and practitioners alike: let us continue to explore, validate, and integrate the immense potential of natural products in the future of internal medicine.
